# Wet-Spun Disulphide LCE Fibres for Continuous Production of Fibrous Artificial Muscles

**DOI:** 10.3390/polym17202789

**Published:** 2025-10-18

**Authors:** Joshua C. Ince, Alan R. Duffy, Nisa V. Salim

**Affiliations:** 1 School of Engineering, Swinburne University of Technology, Hawthorn, Melbourne, VIC 3122, Australia; 2 Space Technology and Industry Institute, Swinburne University of Technology, Hawthorn, Melbourne, VIC 3122, Australia; aduffy@swin.edu.au; 3 Centre for Astronomy and Supercomputing, Swinburne University of Technology, Hawthorn, Melbourne, VIC 3122, Australia

**Keywords:** artificial muscles, polymer actuators, LCE, fibers, wet-spinning

## Abstract

Liquid Crystalline Elastomers (LCEs) are a class of shape-changing polymers with exceptional mechanical properties and potential as artificial muscles/polymer actuators. Much work has been dedicated to expanding the methods available for processing LCEs into various forms using different manufacturing techniques such as 3D printing, film casting, and microfluidic processing. Recently, several works have reported processing LCEs into long fibres and have highlighted the advantages that fibrous LCEs boast over LCE films. However, the development of alternative methods to produce fibrous LCEs is warranted to fully expedite this field of research. In this study, a method for continuous production of disulphide crosslinked LCE fibres via the technique of wet spinning is explored and reported on. Furthermore, the results show that the mechanical properties, actuation force, and actuation strain can be tuned by adjusting how much crosslinker is incorporated into the wet-spinning dope solution. Depending on the given formulation, the reported fibres could repeatedly actuate in response to thermal energy with actuation forces ranging from 0.002 to 0.02 N per fibre and actuation strains ranging from 9.7 to 33%.

## 1. Introduction

The surge in robotics has recently driven the research community to investigate light-weight, low-profile, and flexible solutions to achieve controllable movement [1,2,3]. Typically, moving parts in robotics and machinery are achieved with classical mechanical actuators. However mechanical actuators are heavy, bulky, and rigid. Polymer artificial muscles are classes of polymers that can undergo shape changes and produce work when appropriately stimulated, providing a critical alternative to mechanical actuators [4]. Liquid Crystalline Elastomers (LCEs) are a specific type of polymeric artificial muscles [5]. When stimulated (typically via heating), LCEs contract. When cooled, they are restored to their original shape [6,7,8,9].

LCEs were first reported over four decades ago [10,11,12,13]. Since then, much progress has been made in reporting many different synthetic routes and production methods [14,15,16,17]. However, until recently, LCEs have not been able to truly emulate the functionality and performance of natural muscles. From a characteristic stance, there is considerable overlap between LCEs and natural muscles. Both are anisotropic and both can exhibit large actuation stresses and strains [18]. But natural skeletal muscles are fibrous, and until recently, LCE fibres are not typically reported. LCEs are mostly processed into films but this limits their ability to mimic the actuation of natural skeletal muscles.

Recently, a lot of interest has risen amongst the research community around the use of fibrous LCEs as artificial muscles. Several works have demonstrated the immense potential of fibrous LCEs as artificial muscles [19,20,21]. However, until recently, few works report methods of continuously producing LCE fibres [22].

Roach et al. [23] reported the first continuous and scalable method for producing LCE fibres. By coupling the two-stage Michael addition polymerisation (TMAP) reported by Yakacki et al. [24,25] with the technique of direct ink writing (DIW), the authors reported the first continuous method for producing long LCE fibres. The resultant fibres had actuation stresses of 0.04 MPa and actuation strains of 50% [23]. Following this study, several groups built on this method highlight the true potential of LCE fibres as artificial muscles. Wang et al. [26] used the technique of DIW-TMAP method to produce LCE fibres that were then fashioned into an artificial heart. Sun et al. [27] coated DIW-TMAP produced fibres with liquid metal to form ultra-fast joule-heating LCE fibres. The joule-heating fibres were able to actuate in ~0.1 s at an actuation rate of 280% s^−1^ with actuation strains of 40%. Wu et al. [28] replaced the liquid metal coating with a MXene/polydopamine ink to yield artificial muscles that could lift 1000 times their own mass, actuate in <0.4 s, and could respond to both electrical currents and NIR light. Kim et al. [29] used a melt-extrusion-based modified approach using the DIW-TMAP method to produce expanded graphite-doped LCE fibres that could lift 5000 times their own mass and exhibited a high work capacity of 650 j/kg and a power density of 293 W/kg, significantly outperforming human skeletal muscles. Finally, Hou et al. [30] reported graphene oxide-doped LCE fibres using a modified DIW-TMAP method to produce artificial muscle fibres with an actuation stress of 5.3 MPa, actuation strain rate of ~1000% s^−1^, and ultra-high power density of ~10,000 W/kg.

The forementioned LCE fibre production techniques are indeed promising, and they stand as testimonies to the potential of LCE fibres in artificial muscle applications. However, these works largely rely on the DIW-TMAP method, and, to date, there are few alternative methods to produce LCE fibres. To fully explore the topic of LCE fibres, it is critical to not only explore different LCE formulations but to also explore different fibre production techniques. A common fibre production method is wet spinning [31]. Wet spinning involves extruding a polymer solution into a coagulation bath and then continuously drawing a coagulated polymer fibre from the bath. The benefit of wet spinning as a fibre production method is that it consumes a low amount of energy, can rapidly produce fibres, can align polymer chains in the process of producing said fibres, and is highly scalable. These are attributes that are attractive for the production of LCE fibres; but, aside from a recent article by Martinez et al. [32], wet-spinning LCE fibres is not a thoroughly pursued research topic.

The TMAP chemical approach is the most popular method for synthesising LCEs, and hence, most works exploring processing LCEs into different forms like fibres often rely almost exclusively on the TMAP method. But there are several other alternative synthesis routes for producing LCEs. One such alternative, is to synthesise short thiol end-capped oligomer chains by polymerising diacrylate liquid crystals with flexible dithiols and then employing oxidative decoupling between these oligomers and tri- or tetra-functional thiol compounds, forming dynamic covalent disulphide bond crosslinked networks. This approach was reported by Wang et al. [33] who reported reprogrammable, reprocessable disulphide LCEs. Jiang et al. [34] then reported that this synthetic route can be adapted to 3D-printed disulphide LCE actuators by extruding a precursory disulphide LCE ink into a reactive coagulation bath. As a result of the high reactant concentration in the coagulation bath, the 3D-printed disulphide LCEs were cured in less than 2 s. This prompted the consideration of this approach to be adapted to produce LCE fibres via the technique of wet spinning.

By utilising and adapting the chemistry and methodology reported by Jiang et al. [34], this work reports the characterisation and fabrication of continuously produced wet-spun disulphide LCE fibres. The significance of this work is grounded in the scalability and feasibility of the production method: wet spinning. The reported method allows for the continuous spinning of main-chain LCE fibres. Additionally, because our reported method incorporates disulphide crosslinks, the produced fibres can be aligned by the technique of hot drawing. Finally, the mechanical properties and actuation properties of the produced disulphide LCE fibres were found to be somewhat tuneable by varying how much PETMP is present in the dope formula.

## 2. Materials and Methods

### 2.1. Materials

RM257 was purchased from Suzhou Xiaoli Pharamatech Co., Ltd., Suzhou, China. at a purity of 98.9%. 2,2-(ethylenedioxy) diethanethiol (EDDET), pentaerythritol tetrakis(3-mercaptopropionate) (PETMP), dipropylamine (DPA), and potassium iodide (KI) were purchased from Sigma–Aldrich, Bayswater, Australia. Hydrogen peroxide (35 wv%), tetrahydrofuran (THF), methanol (MeOH), and ethanol (EtOH) were purchased from Chem supply, Gillman, Australia.

### 2.2. Characterisation

Scanning electron microscopy (SEM) imaging was conducted using a Zeiss Gemini SUPRA-40 SEM, Sydney, Australia. Fourier Transformed Infrared Spectrometer was conducted using a Thermoscientific Nicolet 1S50 FTIR spectrometer, Melbourne, Australia. Raman spectroscopy was conducted using a Renishaw InVia Streamline Raman Microscope, Keysborough, Australia. Mechanical analysis and actuation characterisations were conducted using a TA Instruments DHR-1 Discovery Hybrid Rheometer, Rydalmere, Australia. Differential Scanning Calorimetry (DSC) was conducted using a Netzsch Polyma DSC, Brisbane, Australia. Thermo-gravimetric analysis (TGA) was conducted using a TA Q50 TGA, Rydalmere, Australia. ^1^H NMR was conducted using an Ascend 400 Hz Brucker Nuclear Magnetic Resonance (NMR) spectrometer, Preston, Australia, at room temperature using CDCl_3_ as the solvent.

#### Synthesis of Liquid Crystal Oligomer

The thiol end-capped liquid crystal oligomer was synthesised by polymerising RM257 with an excess amount of EDDET in THF. Briefly, 6.0 g of RM257 and 2.4 g of EDDET was dissolved into 20 mL of THF in a 50 mL round-bottom flask. Next 60 µL of DPA was added to the solution and the solution was refluxed at room temperature for 48 h to ensure that the THF did not evaporate and alter the concentration during the polymerisation reaction. Next the solution was placed in a vacuum oven set to 50 °C for 12 h to remove the solvent. The oligomer was then removed from the oven, cooled, and redissolved in 15 mL of THF. The oligomer/THF solution was loaded into a 20 mL syringe and purified using solvent/non-solvent precipitation with 100 mL of MeOH serving as the non-solvent. After the oligomer had coagulated, the MeOH/THF was decanted, and the oligomer was again redissolved in THF. This step was repeated 6 times to purify the synthesised liquid crystal oligomer. Finally, the oligomer was transferred into a weighing vial and placed into a vacuum oven set to 50 °C for 24 h. The yield was determined gravimetrically.

### 2.3. Wet-Spinning Disulphide LCE Fibres

Three solutions were prepared. Each solution contained 1.0 g of the synthesised liquid crystal oligomer and 2.0 mL of THF. Different masses of PETMP equal to 5, 10, and 20 wt% respective to the oligomer mass were added to the solutions. Next, using a micro-pipette, 20, 30, and 80 µL of a 1:1 35 wv% H_2_O_2_:THF were added to the 5, 10, and 20 PETMP mol% solutions, respectively. The solutions were mixed using a vortexer until homogeneous. An amount of 120 µL of a KI catalyst solution was then added to each solution before being mixed again on a vortexer until homogeneous. The catalyst solution was a 2.5 wv% KI solution in 1:3 THF:H_2_O. The wet-spun disulphide LCE (WS-DS-LCE) fibres were then extruded into a 1:3 mL 35 vv% H_2_O_2_:EtOH coagulation bath and wound directly onto rotating drum collectors. The fibres were then placed into a vacuum oven at 50 °C for 24 h to dry.

#### Hot Drawing of Synthesised Wet-Spun Disulphide LCE Fibres

Hot drawing of the synthesised polydomain wet-spun disulphide LCE fibres was conducted using a TA DHR. Fibres were loaded into the DHR tensile geometries, and a small amount of strain was applied to keep the fibres taught. The instrument furnace was then employed to heat the samples to 180 °C for 10 min. The furnace was then opened and the fibres were immediately strained to 100% at a strain rate of 10%/s. The fibres were then left for another 10 min to cool to room temperature before being unloaded from the tensile geometry.

## 3. Results and Discussion

**Figure 1:**
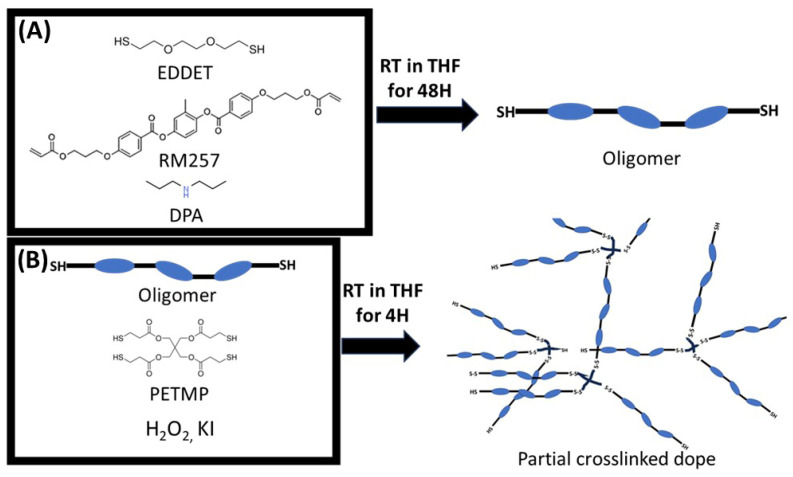
Schematic synthesis of disulphide LCE wet-spinning dope. (**A**) Schematic synthesis of thiol end-capped oligomer. (**B**) Schematic partial crosslinking wet-spinning dope.

Figure 1 depicts the schematic synthesis that was used to prepare the LCE wet-spinning dope. After successfully synthesising the oligomer, the degree of polymerisation (DP) was determined to be 8.89 via ^1^H NMR analysis (Figure S1). The oligomer in our work was determined to be ~2 times the DP compared to the work of Wang and co-authors [33].

Prior to adding the KI catalyst solution, the oligomer and crosslinker solutions were clear and transparent. After adding the catalyst solution, the dopes became slightly yellow and increased in viscosity (see Figure S2).

**Figure 2:**
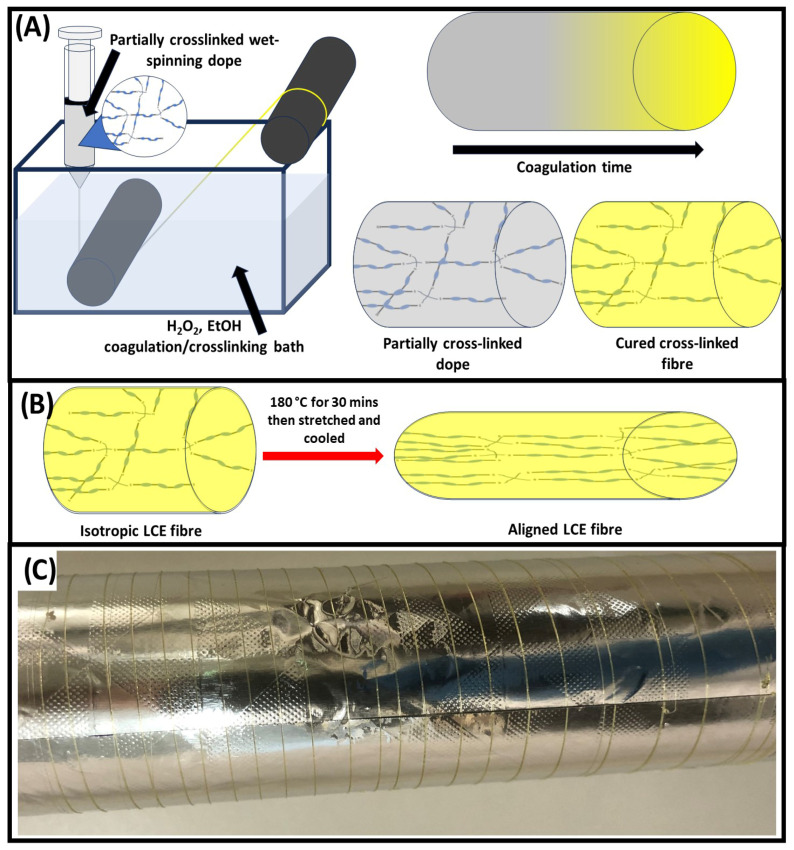
(**A**) Schematic method for wet-spinning disulphide LCE fibres. (**B**) Schematic hot-drawing alignment of wet-spun disulphide LCE fibres. (**C**) Photograph of wet-spun disulphide fibres wrapped around aluminium foil lined collector.

The prepared dopes were then all successfully wet-spun into disulphide LCE fibres. It is important to note that the wet-spinning dope, prior to being extruded into the coagulation bath, remained a solution, albeit a viscous one. This was achieved by controlling the amount of H_2_O_2_ added to the dope solutions such that the LCE network began the process of crosslinking and was sufficiently viscous enough to spin fibres from, whilst remaining below the gel-point, allowing for continuous extrusion of the partially crosslinked dope solution. It is also critical to elucidate that once the partial crosslinking stage was complete, the dope solution was extruded as is. It was not dried and redissolved in solvent as this would have likely caused complications with achieving a homogeneous dope solution due to the decreased solubility of the partially crosslinked network. Figure 2A,B depict the schematic method used to produce and align the wet-spun LCE fibres we report in this work. The high H_2_O_2_ concentration in the coagulation bath, high miscibility of THF, ethanol and water, coupled with the addition of oxidative decoupling catalyst KI in the dope solution, meant that fibres were rapidly solidified, allowing for continuous fibre production. Fibres were able to be continuously collected onto a rotating drum collector, resulting in ultra-long LCE fibres (see Figure 2C for evidence of >1.0 m long fibre). After wet spinning, the fibres turned yellow due to the reaction between KI and the H_2_O_2_ (see Figure 2C). After the WS-DS-LCE fibres were formed, they were able to be hot-drawn, and as shown in Figure S2B, the fibres were observed to be thermally weldable, due to the dynamic covalent disulphide bonds crosslinking the LCE network.

The primary progression that is reported in this paper is the use of the technique of wet spinning to produce LCE fibres. Whilst the DIW-TMAP method for producing LCE fibres is also capable of continuously producing LCE fibres, this method relies on a more sophisticated and complex rig for producing the fibres. For example, in the DIW-TMAP method, a nascent LCE precursor ink is extruded onto a rotating roller. Next, the solvent in the precursor ink must be evaporated so that the extruded fibres have enough mechanical strength to be transferred over to a second rotating roller. Once the solvent in the ink has evaporated, the fibre is wound onto another roller that is rotating at a faster speed than the first roller so that the alignment of the liquid crystal moieties in the LCE fibres can be mechanically induced. To facilitate a feasible solvent evaporation rate so that the fibres can be continuously produced, the first rotating roller must be heated. Additionally, to lock in the induced liquid crystal alignment, right before the LCE fibres are wound onto the second rotating roller, an intense UV light source must be applied to the fibres. Our method utilises coagulation to facilitate the removal of solvent from the precursory LCE dope; hence, no sophisticated heated rotating drum roller is required. The incorporation of dynamic covalent disulphide bonds into our LCE fibres also means that our LCE fibres are aligned via hot drawing, rather than UV curing; hence, no UV-curing system is required in our rig. Alternatively, there are several examples of the wet-spinning LCE fibre method that have been reported in the literature. However, much like the DIW-TMAP method, these works still rely on thiol-ene Michael addition chemistry which almost exclusively necessitates the use of a UV light source to cure the fibres before they are wound onto a rotating collector. Our reported method completely eliminates the need for UV irradiation to continuously produce aligned LCE fibres. In short, our reported approach allows for the continuous production of LCE fibres using nothing more than a syringe pump, a laboratory oven, and requisite chemical reagents, marking a considerable simplification in the production process.

**Figure 3:**
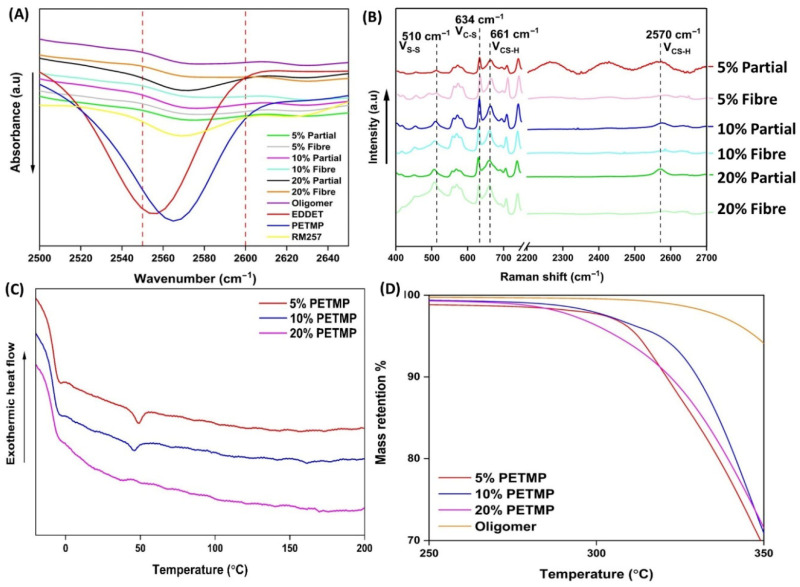
(**A**) FTIR analysis results of WS-DS-LCE fibres, their partially crosslinked dopes, and constituent monomers used to synthesise the dopes. (**B**) Raman spectroscopy results of WS-DS-LCE fibres and their partially crosslinked dopes. (**C**) DSC results of WS-DS-LCE fibres. (**D**) Results to of TGA of WS-DS-LCE fibre and the constituent oligomer.

The complete results of the FTIR spectroscopic analysis are presented in Figure S3, whilst Figure 3A presents the results for the thiol absorption band (2555 cm^−1^). The conducted FTIR analysis alone was found to be inadequate in demonstrating evidence of disulphide bond formation via oxidative coupling. Disulphide bonds did not present in the FTIR analysis due to the lack of a change in the dipole moment when vibrating. Yet the peak attributed to S-H stretching (2500–2600 cm^−1^) in thiol functional groups was expected to diminish in intensity for the wet-spun fibre samples compared to the partially crosslinked dope samples. However, as shown, there was a negligible difference in the peak heights for the partially crosslinked dope samples and the wet-spun fibres.

Figure 3B depicts the acquired Raman spectra for the partially crosslinked wet-spinning dopes and the synthesised disulphide wet-spun LCE fibres. Raman spectroscopy analysis confirmed the formation of disulphide bonds both in the partially crosslinked wet-spinning dopes and in the cured synthesised wet-spun disulphide fibres. The intensity of the peaks attributed to disulphide bonds (ν_S-S_ 510 cm^−1^) in the fibre samples increased, relative to their corresponding partially crosslinked dopes. Concurrently, the peaks attributed to thiol functional groups (ν_CS-H_ 661 cm^−1^ and ν_CS-H_ 2570 cm^−1^) decreased in intensity, providing confirmation of the oxidative coupling of thiol groups to form disulphide crosslinks bonds—a result consistent with the literature. It is noteworthy that the presence of thiol groups was detected in the cured disulphide fibres, providing evidence that the fibres were not completely cured during the wet-spinning process.

The results to the DSC analysis for the synthesised wet-spun disulphide LCE fibres is depicted in Figure 3C. An endothermic phase transition was detected in samples 5% PETMP and 10% PETMP at 50.9 and 44.5 °C, respectively. The same phase transition was not detected in sample 20% PETMP. Some discrepancies exist in the literature as to whether the nematic-isotropic phase transition (T_NI_) can be detected in disulphide LCEs. On one hand, Wang et al. reported that DSC failed to detect the T_NI_ when the group reported the first synthesis of disulphide LCEs [33]. However, Jiang et al. reported the detection of the T_NI_ at 87.2 °C when the group produced disulphide 3D-printed LCE actuators [34]. At the same time, Wang et al. reported that the T_NI_ for the liquid crystal oligomer was ~56 °C, whilst Jiang reported it to be at 76.3 °C. The exothermic peaks detected in samples 5% PETMP and 10% PETMP at 50.9 and 44.5 °C are consistent with the results of Wang et al. and suggest that either un-crosslinked or lightly crosslinked liquid crystal oligomers were present in the fibres. This result is somewhat corroborated by the Raman spectroscopy results. A subtle exothermic peak was observed in all three wet-spun fibre samples at ~70 °C. We suspect that this is the T_NI_; however, the peaks were so subtle that we were not confident in assigning the *T*_NI_ based on these results. The glass transition temperature (*T*_g_) for samples 5% PETMP, 10% PETMP, and 20% PETMP were found to be −10.3, −9.5, and −6.5 °C, respectively. The T_g_ was observed to increase as the PETMP content was increased which is consistent with the literature of disulphide LCEs and the broader field of thermoset polymers [33].

The determined *T*_g_ values are listed Table S1. The disulphide exchange equilibrium temperature was also undetected from the DSC analysis. According to the literature, the exchange should occur at 180 °C. Examples in the literature of disulphide LCEs either do not report DSC analysis at temperatures higher than 150 °C or they do not conduct DSC analysis at all. Therefore, it is difficult to comment on whether the result in this work is anomalous or not.

As depicted in Figure 3D, the TGA results demonstrated that all the samples, including the linear liquid crystal oligomer, were thermally stable up to 250 °C. These results are consistent with other studies on disulphide LCEs reported in the literature. As the crosslinker PETMP content was increased, the thermal stability was seen to decrease. The onset degradation temperatures can be seen to decrease with the increased PETMP content. Furthermore, of all the analysed samples, the oligomer showed the highest thermal stability. Whilst this may seem like an anomalous result, as crosslinking polymers typically lead to an increase in thermal stability, consultation of the literature revealed that this typicality is not always the case. For example, Levchik et al. [35] reported that whilst crosslinked polystyrenes demonstrate an improvement in thermal stability compared to their non-crosslinked counterparts, crosslinked polymethacrylates actually exhibit faster onset of thermal degradation.

Whilst the onset degradation temperatures began for all the samples (except the oligomer) at ~250 °C, the samples were largely stable up to ~270 °C. For example, at 270 °C, less than 5% mass loss had occurred in all the samples. The oligomer sample appeared to be thermally stable at temperatures below 290 °C. However, at temperatures above 300 °C, all samples, including the oligomer, began to degrade much more significantly. The mass loss for all samples, including the oligomer, began to plateau once the temperature passed 450 °C and all samples maintained between 6 and 10% of their mass upon the completion of the analysis. As presented in Table S1, there was no apparent obvious correlation between the final retained mass % after completion of the analysis and the amount of PETMP in the fibres.

**Figure 4:**
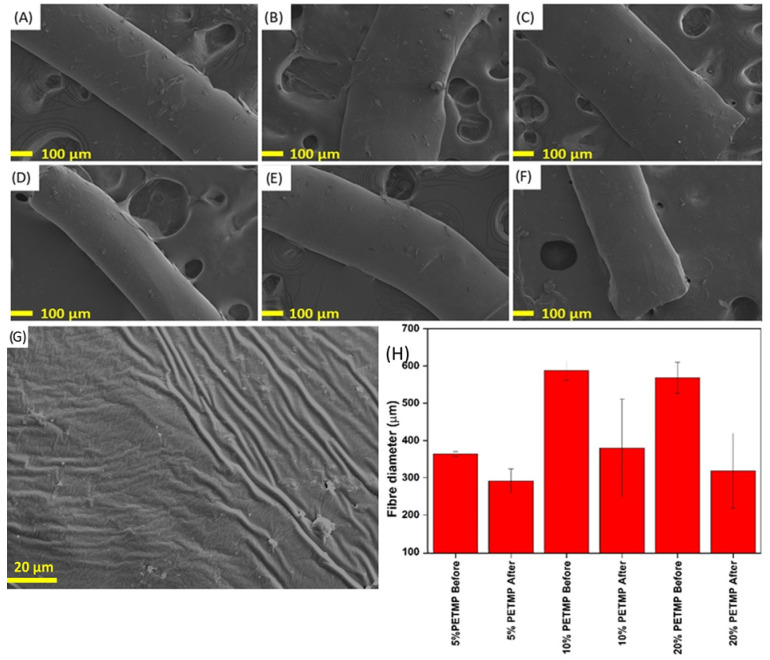
Scanning electron microscopy images of wet-spun disulphide LCE fibres. (**A**–**C**) 5, 10 and 20% PETMP fibres before hot drawing. (**D**–**F**) 5, 10, 20% PETMP fibres after hot drawing. (**G**) SEM image of the surface morphology of 10% PETMP wet-spun fibre. (**H**) Effect of hot drawing on fibre diameter.

SEM images of the synthesised wet-spun disulphide LCE fibres at a magnification of ×100, before and after hot drawing, can be seen in Figure 4A–F. Concurrently, Figure 4H graphically depicts the fibre diameters and the effect of hot drawing on the fibre diameters. There was no observed correlation between the PETMP content and the diameter of the produced fibres. The 10% and 20% PETMP fibres exhibited larger fibre diameters than the 5% PETMP sample. However, this is likely because the fibre production system in this study was not optimised, not because the PETMP content affected the produced fibre diameters. For all samples, hot drawing led to a decrease in fibre diameter, which should be expected given the induced tensile deformation as a result of hot drawing [36]. Prior to hot drawing, the average fibre diameters for samples 5% PETMP, 10% PETMP, and 20% PETMP were 363.7 ± 6.4, 587.9 ± 26.3, and 568.3 ± 41.5 μm, respectively.

After hot drawing, the average fibre diameters were 291.3 ± 32.3, 379.6 ± 130.8, and 318.6 ± 100.5 μm, for samples 5% PETMP, 10% PETMP, and 20% PETMP, respectively. The standard deviation of the fibre diameters considerably increased after hot drawing. We suspect that this result arises from the unoptimised wet-spinning method—the produced fibres did not have uniform diameters prior to hot drawing, and, therefore, the induced strain by the process of hot drawing likely resulted in necking of the fibres, extenuating the fibre diameter deviation [36]. Figure 4G also depicts an SEM image of a piece of the 10% PETMP fibre at an increased magnification of ×2000. As seen in this figure, the surface of the fibre was not porous and, aside from surface wrinkles, was free of visible defects. The observed wrinkles are consistent with the surface morphology of LCEs. Due to their soft, deformable, and elastic mechanical nature, LCEs typically exhibit such wrinkles on the surface. Even in the case of LCE films that are produced by casting a TMAP LCE precursory resin exhibit these wrinkles, and hence, the appearance of said wrinkles on our WS-DS-LCE fibres is typical for LCEs and congruent with the literature [7].

The purpose of this study was to investigate a novel method for producing LCE fibres via wet-spinning that can also be hot-drawn. We noted that several parameters are likely to affect the fibre diameters: namely the gauge of the needle that the dope solution was extruded through, the composition and temperature of the coagulation bath, and the concentration of H_2_O_2_ in the coagulation bath. Additionally, we noted that if we changed the speed of the rotating drum collectors, not only would this have altered the shear forces experienced by the fibres but it would have also altered the time that the fibres spent in the bath which also would have an effect on the fibre diameter—it would be problematic to uncouple the effect between these two variables on the fibre diameter. Hence to simplify the study we kept the drum collector speed, extrusion rate, and needle gauge constant for all the samples and studied the effect of the crosslinker concentration on the fibres mechanical and actuation properties.

**Figure 5:**
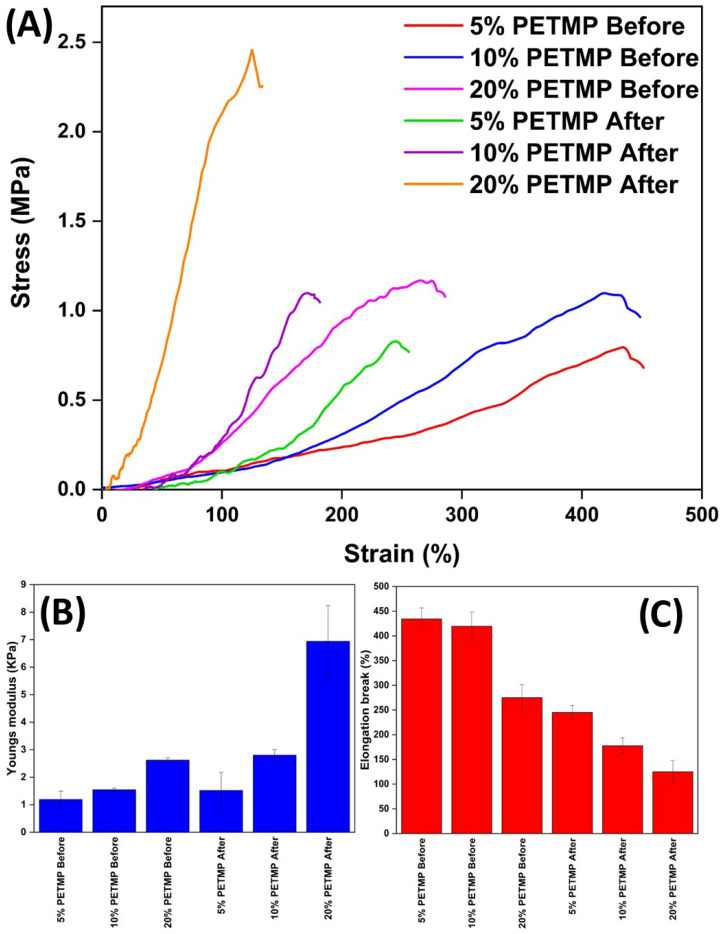
(**A**) Tensile results for wet-spun disulphide LCE fibres before and after hot drawing. (**B**) Young’s Moduli for wet-spun fibres before and after hot drawing, (**C**) Elongation breakage points for wet-spun fibres before and after hot drawing.

The results of the tensile analysis on the wet-spun disulphide fibres before and after hot drawing are depicted in Figure 5A–C. The effect of hot drawing the fibres clearly increased the Young’s moduli for all fibres whilst decreasing the elasticity. This is consistent with other hot-drawn LCEs reported in the literature. The result is due to the alignment of the liquid crystalline moieties within the fibres as a result of hot drawing. Polydomain LCEs are well known to exhibit lower Young’s moduli and to be more elastic than monodomain LCEs. In the case of polydomain LCEs, the initial applied stress gets transferred into aligning the liquid crystal moieties before material necking. For monodomain LCEs, the liquid crystal moieties are already aligned, and hence, once the elastic deformation region is surpassed, the polymers begin to neck sooner than polydomain LCEs. This inherently results in both a higher Young’s modulus and a smaller elastic region in the stress–strain curve. For samples 5% PETMP, 10% PETMP, and 20% PETMP, before hot drawing, the calculated Young’s moduli were 1.19 ± 0.31, 1.55 ± 0.06, and 2.62 ± 0.08 KPa. Concurrently, the elongation breakage points were 434 ± 23, 419 ± 29, and 275% ± 26, respectively. After hot drawing, the Young’s moduli increased to 1.52 ± 0.66, 2.80 ± 0.20, and 6.94 ± 1.31 Kpa for samples 5% PETMP, 10% PETMP, and 20% PETMP, respectively. The elongation breakage points after hot drawing were 244 ± 14, 178 ± 16, and 125 ± 23%, respectively.

Additionally, it was clear from the results that increasing the PETMP content in the produced fibres resulted in a less elastic fibre with a higher Young’s modulus. This trend was observed both before and after hot drawing. This result is also consistent with the literature—increasing the crosslinker content in LCEs results in a higher degree of crosslinking and, therefore, a higher Young’s modulus and a less elastic material.

Comparing the results from this work to that of Jiang et al. [34], the mechanical properties were considerably lower than that of Jiang et al. [34]. This may be due to the incomplete curing of the fibres. Whilst this would explain the discrepancy in mechanical properties, it is a curious result, as Jiang et al. [34] reported that it took <2 s to completely cure their reported disulphide LCEs. Using the same coagulation bath formula, the produced wet-spun disulphide fibres in this study were submerged in the coagulation bath for >2 s. Therefore, it is curious that the fibres were not completely cured. Further studies are necessary to investigate why the fibre sample were not completely cured; we suspect that it may be due to a difference in the concentration of the solvent in the dope solution, resulting in a change in the reaction kinetics and solvent/non-solvent diffusion rates.

**Figure 6:**
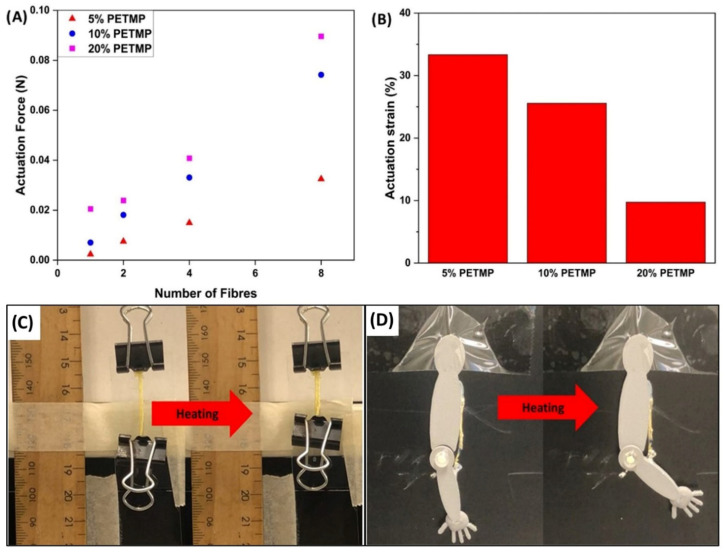
(**A**) Actuation force produced per wet-spun disulphide LCE fibre. (**B**) Actuation strain of wet-spun disulphide LCE fibres. (**C**) Bundle of disulphide wet-spun LCE fibres lifting paper clip 25 times its own mass when heated. (**D**) 3D-printed LCE fibre driven “arm” curling when heated.

To assess the performance of the WS-DS-LCE fibres, DMA was used to assess how much force is produced by a bundle of fibres when the number of fibres is increased. Per Figure 6A, a linear relationship between the number of fibres in a bundle and the actuation force was identified, i.e., a higher fibre count resulted in a higher actuation force. Also, notably, as the PETMP content increased, so too did the actuation force, opening the door for tunable actuation stresses through controlled crosslink density. For context, the calculated actuation stresses from the of the single fibre DMA analysis are featured in Figure 6A. These values were determined to be 0.035, 0.062, and 0.257 MPa, which are higher than the actuation stresses of the DIW-TMAP LCE fibres reported by Roach et al. [23] However, due to the observed and established variability of the fibre diameters, these values may be inaccurate and should be considered as approximate values. Whilst these are impressive values, it should be noted that the WS-DS-LCE fibres reported in this work exhibit actuation strains that are considerably lower than the actuation strains exhibited by LCE fibres using the DIW-TMAP method (~50%) [23]. As depicted in Figure 6B, the actuation strain for the 5% PETMP, 10% PETMP, and 20% PETMP WS-DS-LCE fibres were 33.3, 25.6, and 9.7%, respectively. Evidently, as the crosslinker content increases, the actuation strain decreases, which is a typical and expected effect in LCEs.

From a chemistry perspective, our approach is not novel. The chemistry for the kind of disulphide LCEs utilised in this study was reported in 2017 by Wang et al. [33] Furthermore, in terms of the fundamental phenomenological method, our work is not overly novel. As already discussed, this method was developed by modifying the method reported and outline by Jiang et al. [34] In both the study reported by Jiang and co-authors and in our study, the disulphide LCEs are formed by extruding a partially crosslinked disulphide LCE solution into a reactive coagulation bath to completely cure and solidify the produced LCEs. However, the form is entirely different. Three-dimensional-printed LCEs are certainly a field of research ripe with potential; however, LCE fibres are uniquely biomimetic due to the fibrous nature of natural skeletal muscles. There are a handful of methods that can yield fibrous LCEs; however, this field is still in its infancy stage and most of these methods rely on the TMAP-DIW method or some derivative of this method. The method reported in this work provides an important alternative approach to producing LCE fibres.

To showcase the applicability of the reported wet-spun disulphide LCE fibres as artificial muscles, two demonstrations were designed. As depicted in Figure 6C, a bundle of ×10 10% PETMP fibres could lift a bull clip 200 times its own mass when heated with a heat gun. Next, a 3D-printed “arm” was fabricated and a bundle of ×5 10% PETMP fibres were glued onto the “arm” to act like an artificial bicep muscle (Figure 6D). Through the application of heat via a heat gun, the artificial bicep muscle was able induce bending of the 3D-printed “arm”. These demonstrations can also be seen in Video S1 and Video S2, respectively.

## 4. Conclusions

This work reports a simple and continuous method for producing LCE fibres via the technique of wet spinning. The fibrous morphology of the LCEs in this work mimic the fibrous morphology of natural muscles. The key to this work is the high concentration of H_2_O_2_ in the coagulation bath and the use of KI as an oxidative decoupling catalyst. This allowed for rapid simultaneous coagulation and crosslinking of the wet-spun fibres, enabling the fibres to be easily and continuously wound onto a rotating drum collector. The produced fibres could actuate in response to heat, and the mechanical and actuation properties can be tuned by controlling the amount of crosslinker (PETMP) in the dope solution. Through the utilisation of oxidative coupling of the thiol groups in the constituent oligomer and PETMP, the produced fibres were crosslinked via disulphide bonds. The reversible dynamic covalent nature of disulphide bonds allowed the produced fibres to be aligned via hot drawing, which is atypical for LCE fibres. Furthermore, typical works report that LCE fibres rely on UV irradiation to cure the fibres. The disulphide LCE fibres reported in this work are produced entirely via wet synthetic chemistry.

## Data Availability

The original contributions presented in this study are included in the article/Supplementary Materials. Further inquiries can be directed to the corresponding author.

## Supplementary Materials

The following supporting information can be downloaded at https://www.mdpi.com/article/10.3390/polym17202789/s1, Figure S1: ^1^H NMR results for synthesised liquid crystal oligomer; Figure S2: (A) Precursory partially crosslinked dope solution in glass sample vial. (B) Demonstration of cut WS-DS-LCE fibre being welded back together. (C) Ultralong WS-DS-LCE fibre; Figure S3: FTIR results for produced disulphide LCE fibres, precursor partially crosslinked dopes, and constituent monomers. (A) Complete FTIR spectra for WS-DS-LCE fibres, precursory dopes and constituent monomers. (B) Thiol absorbance region of FTIR results; Table S1: Listing the final retained mass % for synthesized wet-spun disulphide LCE fibres and their constituent oligomer and the detected glass transition temperatures for the synthesised disulphide LCE fibres; Video S1: ×10 bundle of WS-DS-LCE fibres lifting bullclip; Video S2: ×5 bundle of WS-DS-LCE fibres actuating 3D printed arm.
