# Supplementary material for: Wet-Spun Disulphide LCE Fibres for Continuous Production of Fibrous Artificial Muscles
Source: Polymers (Basel). 2025 Oct 18;17(20):2789. doi: 10.3390/polym17202789 (PMC12567026; doi:10.3390/polym17202789)
Supplement: Supplementary file 1 [file polymers-17-02789-s001.zip › polymers-3901623-supplementary.pdf]

Wet-spun disulphide LCE fibres for continuous production of fibrous artificial muscles supporting information

Joshua C Ince [1,3], Alan R Duffy [2,3], Nisa Salim [1, 2, 3] *

[1] School of Engineering, Swinburne University of Technology, Hawthorn, Melbourne, Victoria 3122, Australia

[2] Centre for Astronomy and Supercomputing, Swinburne University of Technology, Hawthorn, VIC, 3122, Australia

[3] Space Technology and Industry Institute, Swinburne University of Technology, Hawthorn, VIC, 3122, Australia

* Corresponding author.

Nisa Salim, Tel: +61 392145703, E-mail: nsalim@swin.edu.au

As seen in Figure S1, ^1H NMR was conducted on the synthesized oligomer to determine the degree of polymerization (DP). The DP was calculated using the method employed by Wang and co-authors [33]. The integration of signals at ~8.15 ppm that were assigned to the protons on the benzene rings in RM257 were set to 4. Next, the signals at 3.6 ppm were assigned to the $-CH_2-$ protons adjacent to the oxygen atoms in EDDET and the integration was determined to be 8.9. The DP was then calculated to be 8/(8.9-8)=8.89.

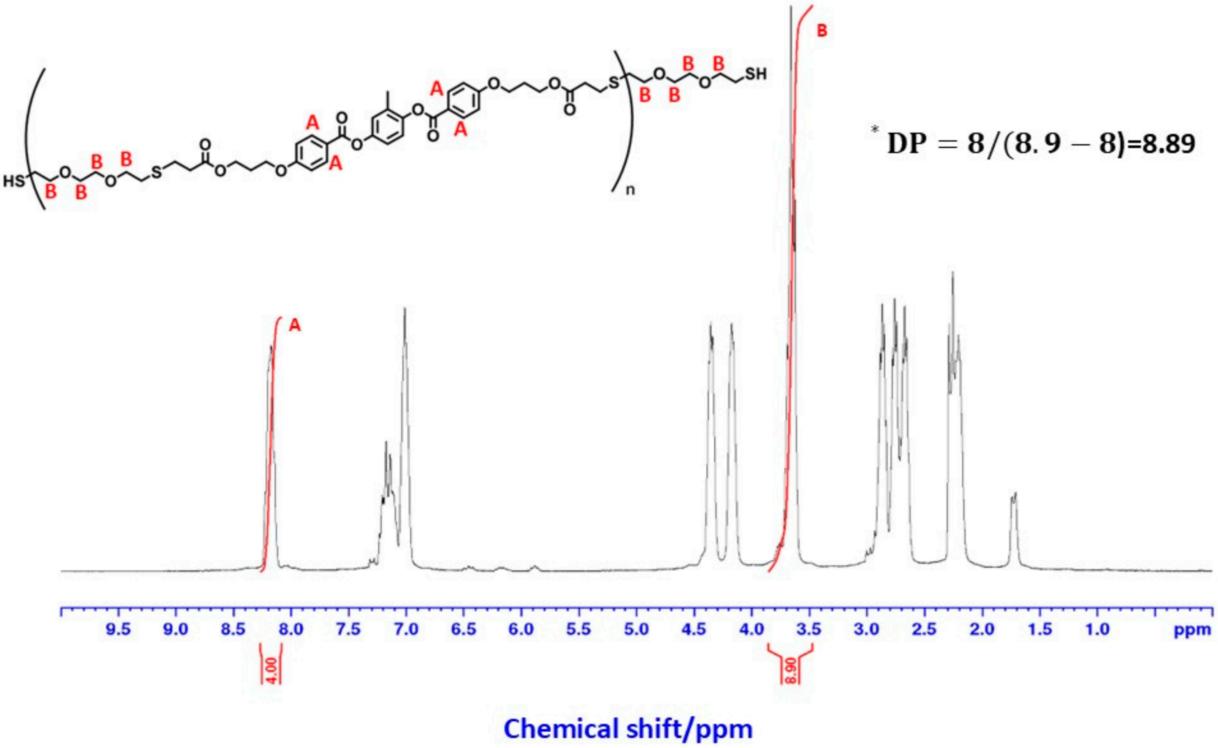

Figure S1. ^1H NMR results for synthesised liquid crystal oligomer.

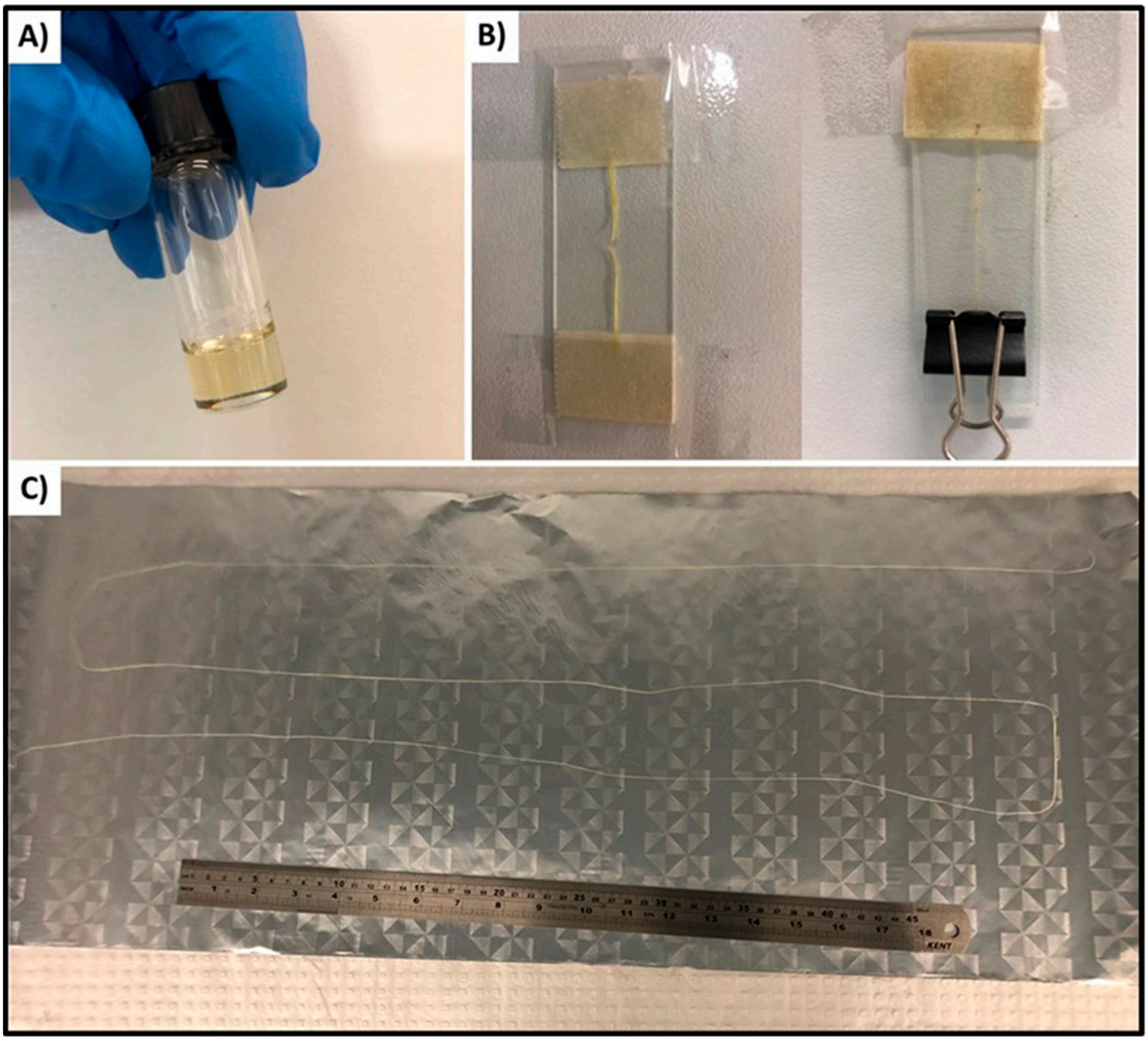

Figure S2.A) Precursory partially crosslinked dope solution in glass sample vial. B) Demonstration of cut WS-DS-LCE fibre being welded back together. C) Ultralong WS-DS-LCE fibre.

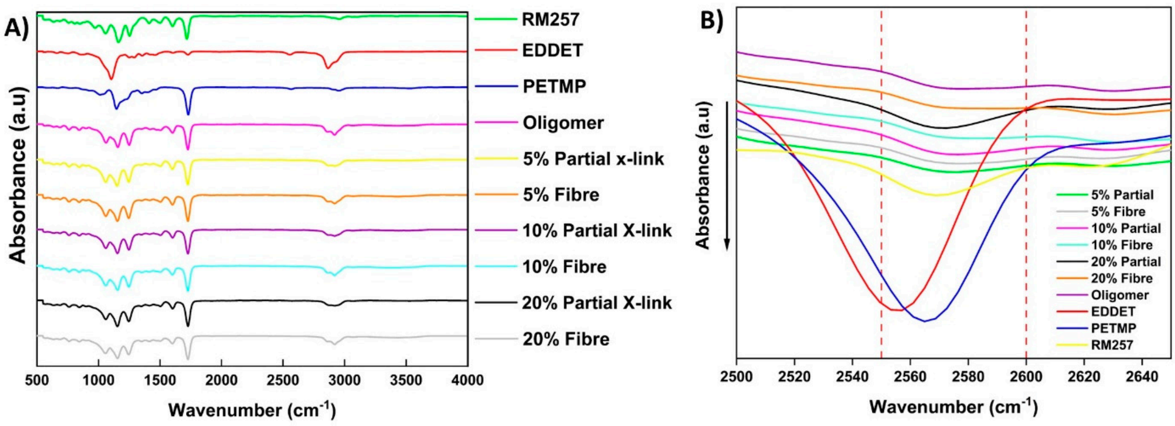

Figure S3. FTIR results for produced disulphide LCE fibres, precursor partially crosslinked dopes, and constituent monomers. A) Complete FTIR spectra for WS-DS-LCE fibres, precursory dopes and constituent monomers. B) Thiol absorbance region of FTIR results.

Table S1. Listing the final retained mass % for synthesized wet-spun disulphide LCE fibres and their constituent oligomer and the detected glass transition temperatures for the synthesised disulphide LCE fibres.

	**5% PETMP**	**10% PETMP**	**20% PETMP**	**Oligomer**
**TGA final retained mass (%)**	6.93	8.51	8.55	9.44
**T_g**	-10.3	-9.5	-6.5	-

It should be noted that at the conception of this study, Small Angle X-ray Scattering (SAXS) or Wide Angle X-ray Scattering (WAXS) was planned to study the alignment of the liquid crystalline moieties in the produced wet-spun disulphide LCE fibres. However, we were unable

to access such characterization techniques. We also noted that conventional powder XRD is not well equipped for characterizing LCEs due to their soft nature and lack of highly ordered crystalline structure. Moreover, in several recent studies X-ray scattering techniques such as SAX or WAXS were not adopted to study the liquid crystalline ordering. As such, we felt the result to our work was still pertinent enough to warrant the release of our findings to the public sphere.
